# Ethics in radiology: A case-based approach

**Published:** 2018-05-31

**Authors:** Laura Stiles-Clarke, James Clarke

**Affiliations:** 1 Faculty of Education, St. Francis Xavier University, Nova Scotia, Canada; 2 Department of Diagnostic Imaging, Dalhousie University, Nova Scotia, Canada

## Abstract

**Background:**

Ethics training is required for all radiology residents in Canada, but this may be difficult to provide as radiology departments may not have radiologists with formal ethics training, and may not have access to educational resources focussed on teaching ethics to radiologists. We describe the implementation of a case-based approach to teaching and learning ethics, designed for Canadian radiologists. This approach can be adapted for use in other specialties through development of specialty-specific ethics case scenarios.

**Methods:**

Ethics case study rounds specific to Canadian radiologic practice were presented at two different institutions, and using two different methods within one institution. In one method, we requested that the residents read the case study and questions ahead of time; in the other, the rounds were presented without any expectation of residents doing prior preparation.

**Results:**

The participants, as a group, agreed with all seven survey statements describing the value of the experience. The opportunity to read the case ahead of time seemed helpful for some residents, but was not found to be overall more useful than discussing the case without prior review. Indeed, more than half of the resident participants in this group indicated that they did not make use of the advance materials at all.

**Conclusion:**

Resident feedback indicates that ethics case study rounds are a useful and valuable experience, especially when the case is specifically tailored to their medical practice. Prior preparation was not necessary for residents to benefit from these rounds.

## Introduction

Ethics education is important for the modern physician, as evidenced by the CanMEDS Professional^1^ role requirement to “demonstrate a commitment to patients by applying best practices and adhering to high ethical standards.”^2^ Unfortunately, medical ethics education can be difficult to provide as part of a residency education program, as discussed by Oljeski, Homer and Krackov:

[Residency] programs may not have faculty who possess formal training in the teaching of ethics. Even if a staff member has taken ethics courses at the undergraduate or graduate level, these courses may not have specifically addressed medical ethics. To compound the problem, we realize that even if the course pertained to medical ethics, the topics may not be relevant to ethics issues that are specific to the practice of radiology.^3^

General medical ethics training is insufficient, as there are a variety of ethical dilemmas in radiology that are unique to the specialty. In addition, “medical ethics is decidedly more interesting and useful to study when it is appropriately tailored to the targeted audience.”^4^

Ethics education is well suited to open-ended, discussion-based teaching, because ethical medical practice is tested in real-life situations as opposed to academic exams. Teaching ethics through lectures can also be problematic due to the unspoken “hidden curriculum”: in managing everyday ethical dilemmas, physicians tend to consult their own moral compasses before, or instead of, recalling what may have been extolled in lectures. Residents then watch and learn from how their staff behave in the real world and are influenced by those behaviours. Our case study rounds were designed to encourage resident engagement and participation, because “residents need to discuss and think about ethics issues in nonthreatening ways before a real, and not theoretic, need to do so arises.”^5^ Shuman, Barnosky, and Koopmann agree that informal sessions work better for ethics education:

Challenging case studies provide a medium that facilitates participation among clinicians who can then hone these skills in an interactive format that is relevant to their practice. Departmental sessions provide an ideal setting whereby colleagues can discuss cases that relate directly to their own experience, while vetting opinions, management options, and perspectives from their coworkers, all within a nonjudgmental forum designed to educate and improve future care.^4^

By presenting an open-ended ethics case study, we intended to encourage such discussions and sharing of experience in a way that lecture-based methods cannot do.

## Methods

### Study design and settings

A set of Canadian radiology-specific case studies was developed collaboratively between the departments of Diagnostic Imaging and Bioethics at Dalhousie University. JC, the second author of this paper and a staff radiologist, developed the case ideas, and a clinical bioethicist from Dalhousie University provided ethical analyses. Each case study presents an ethical challenge based on actual situations that have happened in Canadian radiology practices, and includes an ethical analysis, as well as a set of questions developed by the bioethicist to stimulate thoughtful discussion. The case used in this study has been published^6^ and is available for others to use in their own contexts.

Here, we report on the implementation of ethics case study rounds to radiology residents at Dalhousie University and the University of Alberta, and explore using two different methods of presentation. Prior to these rounds presentations, there was no radiology-specific ethics training provided in either residency program. Residents in both programs would have had a heterogeneous background on non-radiology specific ethics training during their undergraduate and postgraduate medical training.

During rounds, residents were encouraged to place themselves in the shoes of the physician in the case study, and discuss their impressions and what subsequent actions they might take. JC facilitated the rounds, and stated up front that there would be no “correct answer” provided for the case study; the discussion and the participants’ various responses to the case were, in themselves, the purpose of the rounds.

Residents at Dalhousie were presented with two sets of rounds: during the first they were asked to read the case and associated questions before rounds, and during the second they were only presented with the case and questions at the start of the rounds. The intent of these two presentation methods was to determine whether the residents’ experiences of the sessions would be different if participants were given a chance to carefully consider their thoughts before rounds.

A third set of rounds was presented to radiology residents at the University of Alberta with the case and questions being provided ahead of time. Feedback on these rounds was analyzed to see whether the response would be different at a different university.

After each of these sessions, residents were invited to complete a questionnaire regarding the format of the rounds and utility of the rounds to their practice. The questionnaire contained seven statements, which participants rated on a five-point Likert scale (1 meaning “strongly disagree” and 5 meaning “strongly agree”), which was the same approach used by Oljeski, Homer, and Krackov.^3^ Open-ended comments were also solicited. For the rounds where residents had been asked to read the case ahead of time, additional questions were asked about the experience of pre-reading the case.

### Data analysis

All completed questionnaires were analyzed by entering both qualitative and quantitative responses into a Microsoft Excel spreadsheet. Descriptive statistics were used on all quantitative responses and thematic analysis^7^ was carried out with the qualitative responses. The data from the two schools were compared and no significant difference between the two schools was found. Therefore, the data from the two schools were combined in order to create a larger data set.

## Results and discussion

### Quantitative results

Forty-five participants submitted questionnaires for analysis (n=45). For all seven questions, there was a statistically significant difference between combined responses of “strongly agree” or “agree” as compared to combined responses of “neutral,” “disagree,” “strongly disagree,” or no response (Table 1). The mean and standard deviation for the responses to each statement are shown in Table 1.

**Table 1:** Participant responses to feedback statements (n=45)

Statements		strongly agree	agree	neutral	disagree	strongly disagree	no response	Mean ± σ
1	The case study represented a situation that I can foresee facing in my career.	42	2	1	0	0	0	4.91 ± 0.36
2	The discussion questions helped me to develop a response to the case.	27	15	1	1	0	1	4.44 ± 0.94
3	The discussion questions brought issues to light that I hadn’t considered.	15	20	4	4	1	1	3.91 ± 1.16
4	The process of reading the case study and discussing it was valuable to me.	25	17	1	1	0	1	4.47 ± 0.69
5	Hearing the opinions of my colleagues helped me see parts of the case study from a different angle.	30	13	1	1	0	0	4.60 ± 0.65
6	The discussion format of these rounds was better than a lecture format, considering the subject matter.	32	12	0	1	0	0	4.47 ± 0.60
7	The experience of discussing an ethical issue with my colleagues was useful to me as a physician.	31	12	2	0	0	0	4.64 ± 0.57

σ: standard deviation

Participants responded positively to six of the seven questions (Questions 1, 2, 4, 5, 6, and 7) in terms of their Likert scale ratings, as shown by the median values of 5, or “strongly agree”, for these questions. Responses to Question 3 were also positive, although the mean rating for this question was only 3.91 ± 1.16. Question 3, “The discussion questions brought to light issues that I hadn’t previously considered,” was rated lower than the other six survey questions, and its standard deviation was higher, but the participants still agreed with the statement. Although the responses to this question were less positive than the others, the rounds were simultaneously still felt to be beneficial, even by those students who disagreed with this question. This question addressed the discussion questions and not the cases themselves, and the slightly lower response may reflect that the residents had already experienced and thought about that scenario or that they did not believe guiding questions were required to stimulate discussion.

### Qualitative results

A thematic analysis of open-text responses was carried out by LSC, the first author. The themes arising from the data included appreciation for the relevance of the case presented, enjoyment of the small group discussion format, and the desire to hear more cases of this type.

Participants appreciated that the case study had been chosen specifically to benefit their current and future medical practice. Among the many examples of resident comments on the specific relevance of the case are: “Excellent example of a relevant ethical situation,” and “One of the first ethics sessions I found truly applicable to my specialty.” This feedback points to the need for specialty-specific educational resources described by other researchers.^3,4^

The participants also valued the small group discussion, commenting: “Small group discussion was critical, and thoroughly enjoyable!” and “Fun and insightful discussion in small groups. I feel like I actually learned something!”

Several participants stated that they would like to hear more cases of this type. Some of the comments included: “Maybe have more than one scenario,” and “If time permits, discuss additional challenging ethical scenarios.” Two residents requested a short introductory lecture on general ethical principles, and one requested additional references. Another requested a discussion of real-life example cases and their outcomes, similar to the format used by Shuman, Barnosky and Koopmann^4^. Clearly the participants expressed a strong desire to hear further cases tailored specifically to their needs.

More general constructive feedback was also received from all groups of participants. Some examples of these comments are: “Great case with excellent discussion” and “The discussion of what we would actually do vs. the ‘correct’ answer was useful.” No substantial negative feedback was received from any participant.

On the occasions when participants were provided with the case study and questions in advance, they were asked additional questions regarding their experience. Quantitative responses here were inconclusive (Table 2); indeed, of those who were asked to read the case and questions ahead of time, fewer than half claimed to have actually done so. For this reason, we separated the group who stated that they read the materials before the rounds from those who were provided the materials, but did not read them. When comparing these two groups to each other and to the group that was not given the materials ahead of time, in terms of their responses to the first seven questions, we found no significant differences. The qualitative data analysis was similarly inconclusive: no participant commented that the opportunity to read the case ahead of time was detrimental, but some people did find it helpful, and others felt that advance preparation was unnecessary. This may reflect different preferred learning styles, a lack of time to prepare for rounds, or the fact that these rounds required a greater amount of self-introspection rather than memorized facts, as compared to more commonly presented types of rounds.

**Table 2:** Pre-reading responses (n = 32)

	Yes			No		
Did you have a chance to read the case study before rounds today?	15			17		
	**Yes**	**No**	**Not sure**	**Yes**	**No**	**No answer**
Did your ability to read the case ahead of time affect your ability to participate in the discussion?	6	8	1	1	11	5

### Conclusion

Ethics rounds tailored for Canadian radiology residents were developed and presented using two different methods, to two different groups of residents. Quantitative feedback indicated that the participants found the experience to be quite valuable and effective. A thematic analysis of residents’ written comments showed both satisfaction with the ethics case study rounds experience and the desire to participate in further sessions of similar design. No differences were found between groups who had or had not read the preparatory material in advance of the rounds. Future research may include evaluation of faculty satisfaction with case study-based ethics teaching.
